# Assessing Plasmonic Nanoprobes in Electromagnetic Field Enhancement for SERS Detection of Biomarkers

**DOI:** 10.3390/s21248345

**Published:** 2021-12-14

**Authors:** Han-Wen Cheng, Shu-Yan Xue, Jing Li, Justine S. Gordon, Shan Wang, Nina R. Filippone, Quang Minh Ngo, Chuan-Jian Zhong

**Affiliations:** 1 Laboratory of Advanced Materials, Department of Materials Science, Fudan University, Shanghai 200438, China; shuyan_xue@fudan.edu.cn; 2 Department of Chemistry, State University of New York at Binghamton, Binghamton, NY 13902, USA; jli182@binghamton.edu (J.L.); jgordo35@binghamton.edu (J.S.G.); swang129@binghamton.edu (S.W.); nfilipp1@binghamton.edu (N.R.F.); 3 Vietnam Academy of Science and Technology, University of Science and Technology of Hanoi, 18 Hoang Quoc Viet, Cau Giay, Hanoi 11307, Vietnam; quangminhims@gmail.com; 4 Institute of Materials Science, Vietnam Academy of Science and Technology, 18 Hoang Quoc Viet, Cau Giay, Hanoi 11307, Vietnam

**Keywords:** gold nanoparticles, magnetic core–shell nanoparticles, plasmonic nanoprobes, SERS detection, cancer biomarker, electromagnetic field enhancement

## Abstract

The exploration of the plasmonic field enhancement of nanoprobes consisting of gold and magnetic core@gold shell nanoparticles has found increasing application for the development of surface-enhanced Raman spectroscopy (SERS)-based biosensors. The understanding of factors controlling the electromagnetic field enhancement, as a result of the plasmonic field enhancement of the nanoprobes in SERS biosensing applications, is critical for the design and preparation of the optimal nanoprobes. This report describes findings from theoretical calculations of the electromagnetic field intensity of dimer models of gold and magnetic core@gold shell nanoparticles in immunoassay SERS detection of biomarkers. The electromagnetic field intensities for a series of dimeric nanoprobes with antibody–antigen–antibody binding defined interparticle distances were examined in terms of nanoparticle sizes, core–shell sizes, and interparticle spacing. The results reveal that the electromagnetic field enhancement not only depended on the nanoparticle size and the relative core size and shell thicknesses of the magnetic core@shell nanoparticles but also strongly on the interparticle spacing. Some of the dependencies are also compared with experimental data from SERS detection of selected cancer biomarkers, showing good agreement. The findings have implications for the design and optimization of functional nanoprobes for SERS-based biosensors.

## 1. Introduction

Surface-enhanced Raman spectroscopy (SERS) is a powerful technique for bioassays with high sensitivity, high selectivity, and a low detection limit, which stems largely from the strong dependence of signal amplification. Nanomaterials consisting of gold or silver nanoparticles and nanoparticles with magnetic cores and gold or silver shells have found increasing applications as an intriguing class of SERS substrates or probes to produce a strong plasmonic resonance enhancement [1,2]. There has been significant progress in harnessing SERS nanoprobes for biosensors [1], but the understanding of the factors controlling the plasmonic field enhancement remains elusive. This understanding is particularly important in view of the increasing application of SERS in cancer biomarker detection [2], which shows intriguing attributes (e.g., much lower LOD) in comparison with traditional techniques, such as enzyme-linked immunosorbent assay (ELISA) and radio immunoassay, that are typically time-consuming and complex. Cancer biomarkers (protein, DNA, etc.) are released from cancer cells, and their detection has applications in medical diagnostics and therapies [3,4,5,6,7,8]. A simple and effective strategy involves exploring Au nanoparticles (NPs) and magnetic core–gold shell NPs for effective coupling of magnetic and plasmonic properties in SERS detection of DNAs and cancer biomarkers [4,9,10,11]. In addition to the plasmonic properties [12,13,14], the magnetic properties enable separation and enrichment in signal amplification [8,12]. In SERS-based immunoassays, this magnetic enrichment can be coupled with the plasmonic resonance enhancement due to the antibody–antigen bonding affinity in between the NPs, leading to “hot-spot” SERS signal amplification. For example, magnetic MnZn ferrite NPs decorated with Au or Ag atoms or shells on the surface (M@Au or M@Ag) function as effective nanoprobes for SERS detection of double-strand DNA (*ds*DNA)-linked M@Au or M@Ag NPs and Au NPs [4,15]. The “hot-spot” electromagnetic field enhancement of the *ds*DNA-linked NPs was also supported by theoretical simulation [16] in terms of the effective thickness of DNA layers on an NP dimer model. The Au and magnetic core@Au shell strategy has also been demonstrated to be viable for SERS and electrochemical detection of anticancer drug interactions with DNAs [17,18]. Overall, significant progress has been made in developing different SERS substrates, including plasmonic metal nanoparticles for detection of various biomolecules [1], and other self-assembled 2D nanomaterials such as transition metal containing carbides, nitrides, or carbonitrides for detection of various molecules [19,20].

**Figure 1:**
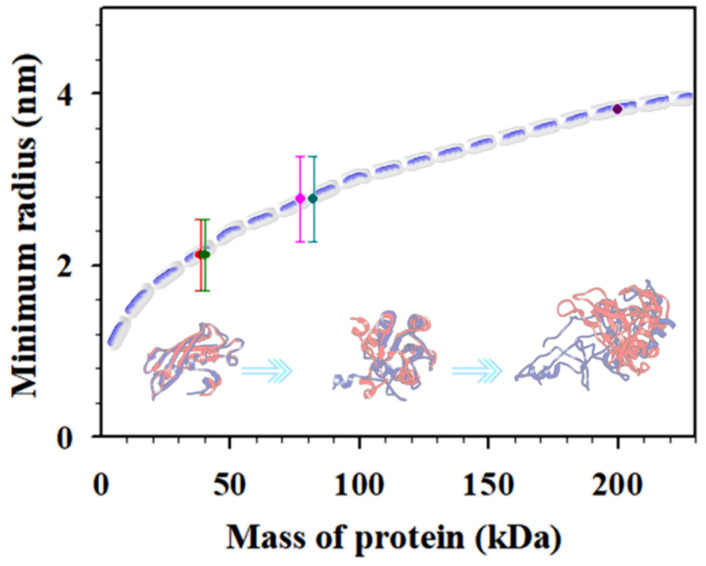
Plot of the minimum radius for proteins of different masses. The data points were estimated for NSE (monomer, 38.5 kDa), soluble fragment of cytokeratin 19 (CYFRA21–1, ~40 kDa), neuron-specific enolase (NSE, dimeric, 77 kDa), carbohydrate antigen 15–3 (CA 15–3, 82 kDa), and CEA (200 kDa). Inset: illustrative scheme of different protein masses.

Plasmonic nanoparticle nhanced “hot-spots” have been exploited for SERS detection of cancer biomarkers [2] including carcinoembryonic antigen (CEA), alpha fetoprotein (AFP), human epidermal growth factor receptor 2 (HER2), vascular endothelial growth factor (VEGF), prostate-specific antigen (PSA), tumor suppressor p53, epidermal growth factor receptor (EGFR), and neuron-specific enolase (NSE) [1,21]. We recently demonstrated the combination of Au and magnetic core@Au shell nanoprobes for SERS detection of CEA via conjugation of the nanoprobes with CEA-specific detection and capture antibodies [9]. A similar strategy has also been used for simultaneous detection of CEA and NSE in buffer and human serum, showing good specificity, high sensitivity, and low detection limits (LOD for CEA (1.48 pg/mL) and LOD for NSE (2.04 pg/mL)) [22]. The flower-like gold nanoparticles (~748 nm) were conjugated with anti-CEA and anti-NSE antibodies separately, and gold-functionalized magnetic Fe_3_O_4_ nanoparticles (~160 nm) were conjugated with both anti-CEA antibody and anti-NSE antibody. These nanoprobes were magnetically concentrated on a substrate with a high density for the SERS detection. Despite the progress, a fundamental question for the “hot-spot” detection strategy is how the plasmonic field enhancement depends on nanostructural parameters such as particle core size, shell thickness, and, more importantly, the antigen/antibody sizes. In this work, we further studied the “hot-spot” characteristics based on a gold and core–shell NP dimer model [4] using various combinations of magnetic core–gold shell nanoparticles in the detection of cancer biomarkers (CEA, NSE, etc.). The size of protein molecules is related to the molecular mass (see Figure 1, dashed line) [23]. Assuming close packing of proteins (1.37 g/cm^3^), the minimum radius of the protein, R_min_, calculated [2,23] gives, e.g., CEA (200 kD) ~3.8, CYFRA21−1 (~40 kD) ~1.8~2.4, NSE (dimeric 77 kD) ~2.4~3.1, NSE (monomer 38.5 kD) ~1.8~2.4, and CA 15−3 (82 kD) ~2.4~3.1 nm, ranking CEA > CA15−3 > NSE > CYFRA21−1 (see Figure 1, dots). The minimum radius for proteins of different mass scales with molecular mass are shown in Figure 1. The variation of the interparticle parameter upon their bioconjugation onto the nanoprobes tunes the “hot-spot” SERS signals.

In this report, we expanded our recent simulation work on nanoparticle-based SERS detection of DNAs [3] to assess the plasmonic field enhancement of gold and magnetic core@gold shell nanoparticles in terms of antigen- and antibody-defined interparticle spacing and nanoparticle structural parameters. A novel aspect of this present work was to provide correlation of the core–shell-type nanoparticles with magnetic cores of different sizes and gold shells of different thicknesses for interparticle binding of antigens/antibodies of different sizes with the EM-field enhancement. The results were also compared with experimental data from nanoparticle-based SERS detection of cancer biomarkers (see related Experimental details and Simulation details in the Supplementary Materials).

## 2. Results and Discussion

### 2.1. The Dimer Model and the Simulated Electromagnetic Field Intensity (EMF)

MNPBEM toolbox was used to perform the simulation of the electromagnetic field (EMF) intensity as a result of the localized surface plasmon resonances of the NPs in an aqueous environment (see details in Supplementary Materials) [3,16]. In the MNPBEM toolbox, the optical constants of the nanoparticles were used in the Maxwell equations for the simulation. The dielectric constants for gold and magnetic iron oxide were used in the simulation [3]. The wavelength of incident light was 780 nm.

The simulations were based on dimer models in terms of the electromagnetic field intensities with spherical gold and magnetic core–gold shell nanoparticles [3,24] such as the dimer model of nanoparticles formed via capture antibody (Ab1)–antigen detection antibody (Ab2) binding. The nanoparticles could be Au nanoparticles, magnetic nanoparticle (MNP) cores with Au shells (i.e., M@Au), or their combination with different sizes or shell thicknesses. The Au-based surface allows labeling or conjugation of the Raman-active labels or the bio-active antibodies (Ab1 or Ab2). Examples of MNPs include Fe_3_O_4_ [25,26], MnZnFeO_2_ [4,27,28], and NiFe NPs [9]. The NiFe NPs used for the synthesis of M@Au NPs featured Fe_3_O_4_ on the surface of the NPs [9,29].

The simulation was based on a dimer model of the nanoparticles of various sizes via capture antibody–antigen–detection antibody binding. The plasmonic field under an electromagnetic field depends on the locations in the dimer, e.g., Au_O_, at the outer-edge of Au NP; Au_I_, at the inner-edge of Au NP; C, at the center of the antigen; M@Au_I_, at the inner-edge of magnetic core@shell NP (Fe_3_O_4_ core-Au shell); M@Au_O_, at the outer-edge of core@shell NP.

In this model, the coupling of the surface plasmon resonances led to electromagnetic filed enhancement depending on several parameters including the size of the Au NPs, size of the magnetic cores, thickness of the Au shells, and the interparticle spacing defined by the antibody–antigen–antibody (i.e., Ab1–Antigen–Ab2) binding in between the NPs. By varying these parameters, the electromagnetic or plasmonic fields around the NPs and between the NPs were calculated [3]. The selected results are described in the following subsections, providing information for assessing the plasmonic enhancement around the NP or between the NPs.

**Figure 2:**
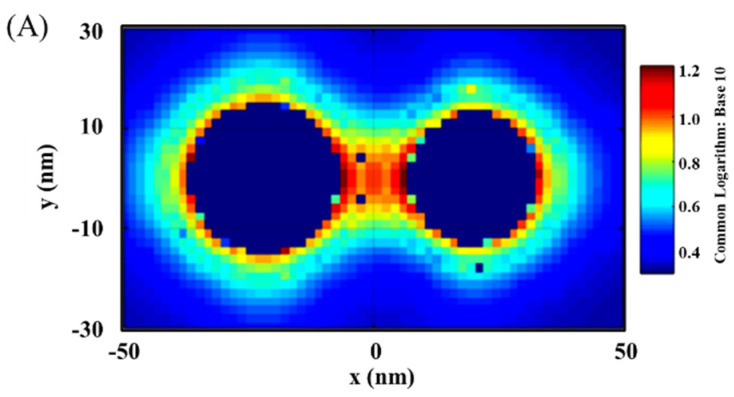

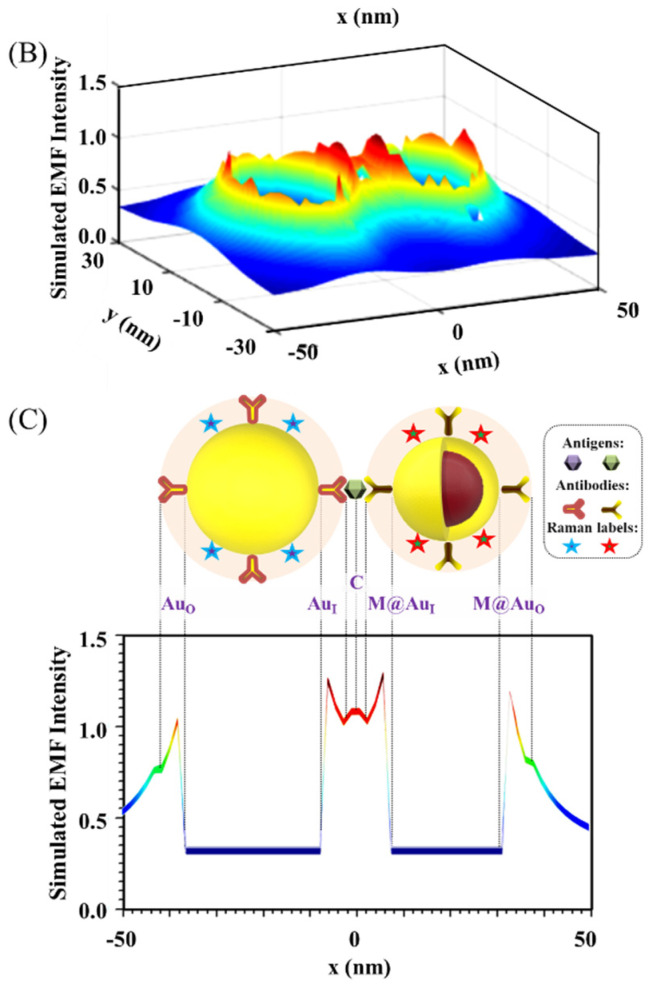
EMF in 2D mapping (**A**), 3D mapping (**B**), and 2D plotting at y = 0 (**C**). The inset in (**C**) is the illustration of the dimer model: Ab1_4.8 nm_/Au_30 nm_–NSE_4.2 nm_–Ab2_4.8 nm_/M@Au_26 nm_ (M@Au NP: 6 nm Fe_3_O_4_ core and 10 nm Au shell). Au_O_, at the outer-edge of Au NP; Au_I_, at the inner-edge of Au NP; C, at the center of the antigen; M@Au_I_, at the inner-edge of magnetic core@shell NP (Fe_3_O_4_ core-Au shell); M@Au_O_, at the outer-edge of core@shell NP.

Figure 2 shows a typical set of results showing the distribution of the plasmonic fields in terms of the intensity in 2D/3D mapping. As shown in Figure 2A,B for the plasmonic filed gradient around the NPs, there was clear intensification near the surface of the nanoparticles and in the space between the two nanoparticles (hot spots). The E-field intensification was further analyzed in Figure 2C by plotting the field vs. distance at y = 0. In this plot, some of the strong E-field intensity locations can be clearly identified on the edges of the NPs or the center between the NPs.

It is evident that the EMF intensity depended strongly on the locations as visually illustrated in the top panel of Figure 2C. For the convenience of discussion in terms of the locations, we labeled several representative locations in reference to the interparticle region. Three of the major locations inside the interparticle region include the inner-edge of Au NP (Au_I_), the inner-edge of magnetic core@Au shell NP (M@Au_I_), and the edge-to-edge center of the interparticle region (C). Two of the major locations outside the interparticle region include the outer-edge of the Au NP (Au_O_) and the outer-edge of magnetic core@Au shell NP (M@Au_O_). In the following subsection, we focus on analyzing the trends of the EMFs at these different locations as illustrated in Figure 2C, i.e., Au_O_, Au_I_, C, M@Au_I_, and M@Au_O_. These locations give an overall picture of the EMFs in the dimer model in relation to the plasmonic field enhancement.

### 2.2. EMFs in Correlation with the Nanostructure Parameters

The EMF intensities at the specified locations in the dimer models were analyzed by varying the Au NP sizes, MNP core sizes, Au shell thicknesses, and the antibody–antigen–antibody binding defined interparticle spacing.

#### 2.2.1. Au NP Size

**Figure 3:**
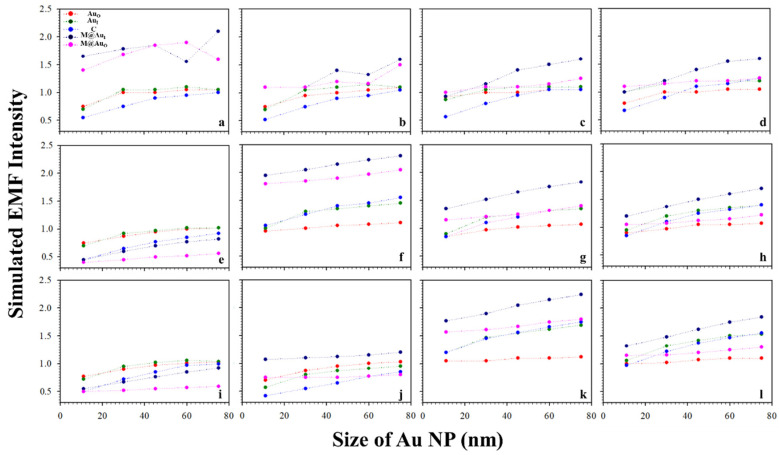
Plots of the simulated EMF intensity for the dimer of “Ab1/Au–CEA–Ab2/M@Au” vs. Au NP size at different locations at the outer-edge of the Au nanoparticles (Au_O_, red circle), inner-edge of Au nanoparticle (Au_I_, green circle), the center in-between (C, blue circle), inner-edge of MNP (Fe_3_O_4_ core–Au shell) (M@Au_I_, dark blue circle), and outer-edge of MNP (M@Au_O_, pink circle). (**a**–**d**) 6 nm Fe_3_O_4_ core–Au shell thickness of 1 (**a**), 3 (**b**), 5 (**c**), and 10 nm (**d**); (**e**–**h**) 20 nm Fe_3_O_4_ core–Au shell thickness of 1 (**e**), 3 (**f**), 5 (**g**), and 10 nm (**h**); (**i**–**l**) 30 nm Fe_3_O_4_ core–Au shell thickness of 1 (**i**), 3 (**j**), 5 (**k**), and 10 nm (**l**).

It is known that the surface plasmon resonance (SPR) band of Au NPs depend on the particle size, which is reflected by both the SPR band position and intensity and SERS peak intensity [2]. Figure 3 shows a typical set of plots of the EMFs at the different locations (Au_O_, Au_I_, C, M@Au_I_, and M@Au_O_) of the dimer with various magnetic core sizes (6, 20, and 30 nm) and Au shell thickness (1, 3, 5, and 10 nm) vs. Au NPs size (11, 30, 45, 60, and 75 nm). The interparticle distance was defined by antibody–antigen–antibody binding (Ab1/Au–CEA–Ab2/M@Au). Note that the lengths for both Ab1 and Ab2 were 4.8 nm, and the length for CEA was 7.6 nm [2,23]. The EMF intensities were plotted vs. Au NP size at different locations; Au_O_ (red circle), Au_I_ (green circle), C (blue circle), M@Au_I_ (dark blue circle), and M@Au_O_ (pink circle). In general, the EMFs at Au_O_, Au_I_, C, M@Au_I_, and M@Au_O_ increased with the size of Au NPs, showing subtle differences in terms of the absolute values. The increase in EMF intensities was intensified at M@Au_I_ and M@Au_O_ with the increase in Au shell thickness. However, there were some cases where EMFs showed sharp overall increases, as in Figure 3 (core_6 nm_−shell_1 nm_ (a), core_20 nm_-shell_3 nm_ (f), and core_30 nm_-shell_5 nm_ (k)), in comparison with the EMF intensities of the same core size but different Au shell thickness. This indicates that the EMF enhancement strongly depended on the detailed core–shell combination, the understanding of which requires further investigation.

**Figure 4:**
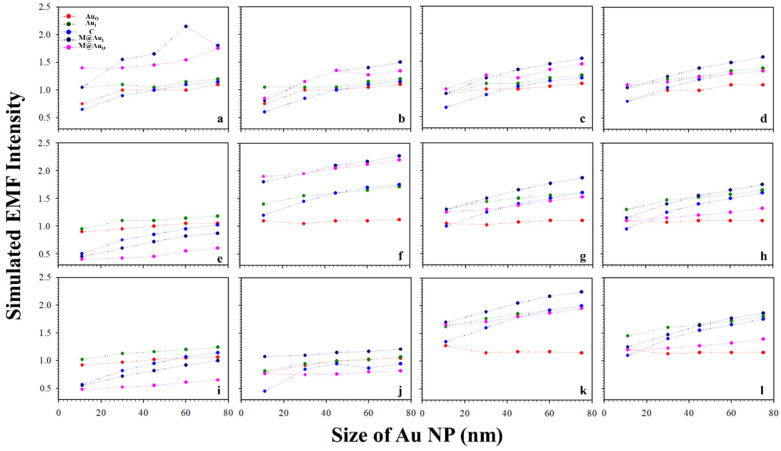
Plots of the simulated EMF intensity for the dimer of “Ab1/Au–NSE–Ab2/M@Au” vs. Au NP size at different locations of Au_O_, Au_I_, C, M@Au_I_, and M@Au_O_. (**a**–**d**) 6 nm Fe_3_O_4_ core–Au shell thickness of 1 (**a**), 3 (**b**), 5 (**c**), and 10 nm (**d**); (**e**–**h**) 20 nm Fe_3_O_4_ core–Au shell thickness of 1 (**e**), 3 (**f**), 5 (**g**), and 10 nm (**h**); (**i**–**l**) 30 nm Fe_3_O_4_ core–Au shell thickness of 1 (**i**), 3 (**j**), 5 (**k**), and 10 nm (**l**).

Similar trends were observed for a different interparticle distance, e.g., a distance defined by “Ab1/Au–NSE–Ab2/M@Au”. Figure 4 shows a typical set of plots of the plasmonic fields at the different locations (Au_O_, Au_I_, C, M@Au_I_, and M@Au_O_) of the dimer with various magnetic core sizes (6, 20, and 30 nm) and Au shell thickness (1, 3, 5, and 10 nm) vs. Au NPs size (11, 30, 45, 60, and 75 nm). In general, the trends were very similar to those in Figure 3 with subtle differences in absolute values. Again, similar sharp increases in EMF were also observed in Figure 4 for several different core–shell combinations, including core_6 nm_–shell_1 nm_ (a), core_20 nm_–shell_3 nm_ (f), and core_30 nm_–shell_5 nm_ (k). Moreover, the overall EMF values in Figure 4 were slightly higher than those with the corresponding Au NP sizes and core–shell sizes/thicknesses in Figure 3. In comparison with the case for CEA (Figure 3), the overall EMF intensities were greater in the case of NSE (Figure 4), demonstrating the strong dependence of EMF on the interparticle spacing.

#### 2.2.2. MNP Core Size

**Figure 5:**
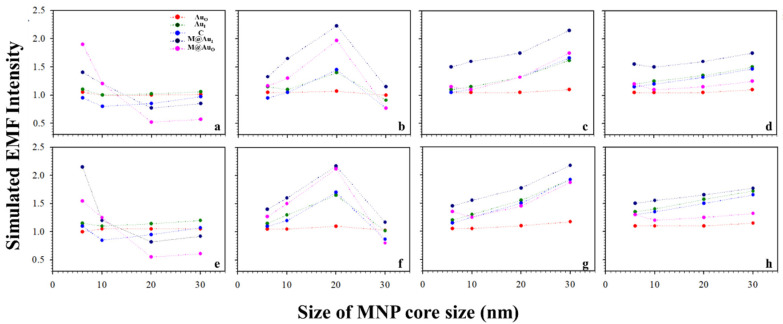
Plots of the simulated EMF intensity for the dimer of “Ab1/Au_60 nm_–CEA (**a**–**d**) or NSE (**e**–**h**)–Ab2/M@Au” vs. different sizes of Fe_3_O_4_ core at different locations of Au_O_, Au_I_, C, M@Au_I_, and M@Au_O_. Different Fe_3_O_4_–Au shell thicknesses of 1 (**a**,**e**), 3 (**b**,**f**), 5 (**c**,**g**), and 10 nm (**d**,**h**).

Based on the above results, the plasmonic field intensity was further plotted as a function of the MNP core sizes. Figure 5 shows the simulated EMF intensity for the nanoparticle dimer of CEA (a–d) or NSE (e–h) vs. the size of the Fe_3_O_4_ core. Interestingly, as the core size increased, the EMF decreased for small Au shell thicknesses (<4 nm, a and e), and it reached a maximum before decreasing for an intermediate shell thickness (b and f). It increased for large shell thicknesses before diminishing the trend (>3 nm, c–d and g–h). This type of trend was observed for both the Ab1–CEA–Ab2 and Ab1–NSE–Ab2 cases. Note that little change was observed for Au_O_ with the change in shell thickness. This may be understood by the limited plasmonic field of this location being far away from the strong plasmonic field in the interparticle region.

#### 2.2.3. Au-Shell Thickness

**Figure 6:**
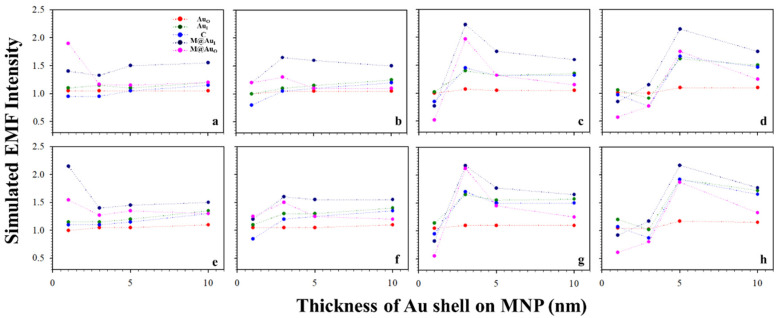
EMFs vs. Au shell thickness on MNP with the interparticle distances defined by Ab1–antigen–Ab2 binding. Plot of the simulated EMF intensity for the dimer of “Ab1/Au_60 nm_–CEA (**a**–**d**) or NSE (**e**–**h**)–Ab2/M@Au” vs. different thicknesses of Au shells on MNPs at different locations of Au_O_, Au_I_, C, M@Au_I_, and M@Au_O_. Different sizes of Fe_3_O_4_ cores of 6 (**a**,**e**), 10 (**b**,**f**), 20 (**c**,**g**), and 30 nm (**d**,**h**).

Figure 6 shows the plots of the simulated EMF intensity for the dimer of CEA (a–d) or NSE (e–h) using Ab-conjugated Au NPs_60 nm_ vs. different thicknesses of Au shell on MNP. The EMF changes were relatively small for the small MNP core sizes (<10 nm). However, it increased with shell thickness for larger core sizes (>10 nm), showing a maximum EMF at a shell thickness of ~5 nm (d and h). Again, little change was observed for Au_O_ with the change in shell thickness, which is consistent with the results in Figure 5.

#### 2.2.4. Interparticle Spacing

**Figure 7:**
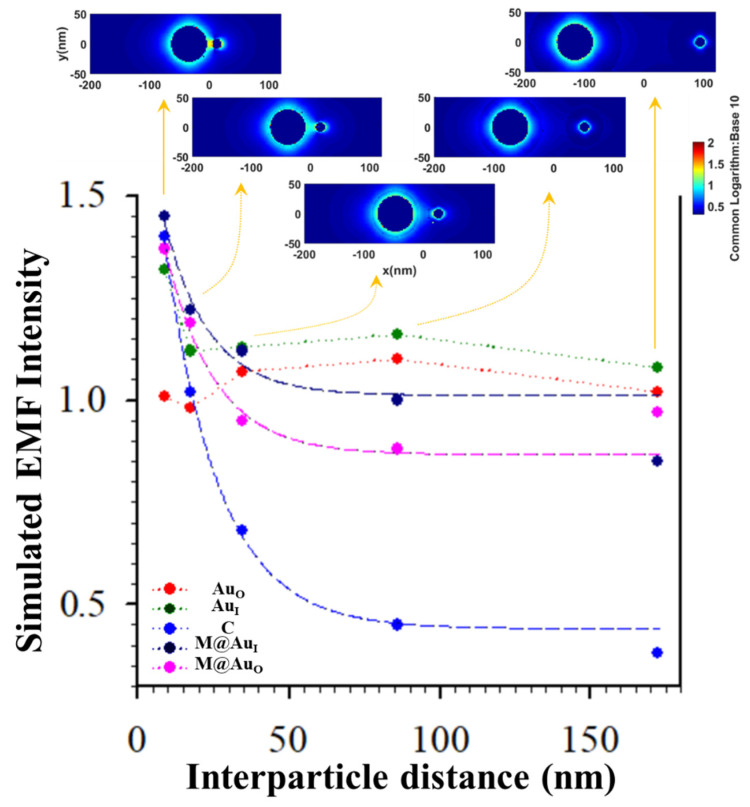
Plots of EMFs vs. interparticle distance for the dimer of “Ab1/Au_60 nm_–antigen–Ab2/M_6 nm_@Au_5 nm_ NP” at the different locations of Au_O_, Au_I_, C, M@Au_I_, and M@Au_O_. Inset: plasmonic field simulation results in terms of 2D plotting. Data were fitted by an exponential decay model yielding: y = 0.4412 + 1.5384 × e^−0.0556x^ (C, blue), y = 1.0123 + 0.7650 × e^−0.0679x^ (M@Au_I_, dark blue), and y = 0.8671 + 0.8674 × e^−0.0615x^ (M@Au_O_, pink) (see Figure S1 (Supplementary Materials) for plots of average EMFs near y = 0 (±5 nm)).

Figure 7 shows the plot of EMF intensity for the dimer of “Ab1/Au_60 nm_–CEA–Ab2/M-core_6 nm_@Au_5 nm_ NP” vs. the interparticle distance (*d*) defined by the antibody–antigen–antibody binding. The EMF intensities at the location of C (blue circle) showed a clear trend of gradual decrease vs. interparticle spacing *d*. Interestingly, the strongest EMF locations around the two NPs exhibited a similar trend of the gradual decrease. Data in Figure 7 are fitted by an exponential decay model as a function of the interparticle distance (exp(-*kd*)), from which *k* value for EMF location-C (0.0556) was 82% of that for location-M@Au_I_ (0.0679) and 90% for location-M@Au_O_ (0.0615). Note that there were subtle increases for Au_O_ and Au_I_ which were observed at *d* < 35 nm. To understand this, we closely examined the EMFs in the region near y = 0 (±5 nm) to cover the interparticle zone. The average values of EMFs in this zone vs. *d* plots are shown in Figure S1 (see Supplementary Materials). While the overall trends of the EMF intensity remain unchanged, a subtle increase for the location of M@Au_O_ was observed at *d* < 35 nm, similar to the subtle increase for the locations of Au_O_ and Au_I_. While the exact origin of this subtle increase is unclear, we believe that these subtle differences reflect the differences in the sensitivity of the EMFs to the interparticle plasmonic coupling at the various locations in the dimer. The closer the location to the interparticle center, the more sensitive the EMF to the change in the interparticle distance. The smaller the value of *k*, the more sensitive the EMF at the location to the interparticle distance. While EMF at location-C may not be the strongest depending on the particle sizes and core–shell nanostructures, it is evident that the EMF at location-C was the most distance-sensitive among all locations. This finding is consistent with the maximization of plasmonic coupling of the nanoparticles at the interparticle center of the dimer model.

To further substantiate the above assessment, we examined dimers consisting of only Au NPs (i.e., without M@Au NP). Figure S2 (see Supplementary Materials) compares EMF intensities for the dimers of “Ab1/Au_60 nm_–CEA or NSE–Ab2/Au_16 nm_” (a) and “Ab1/Au NP_60 nm_–CEA or NSE–Ab2/Au_60 nm_” (b) for the two different interparticle distances, i.e., “Ab1–CEA–Ab2” (17.2 nm) and “Ab1–NSE–Ab2” (13.8 nm), at Au_O_, Au_I_, C, control-Au_I_, and control-Au_O_. The results are consistent with those of the M@Au NPs in terms of the sensitivity of the EMF at the different locations to the change in interparticle distance. The most sensitive location was the particle edge-to-edge center (C). The next most sensitive locations are those closest to the center (i.e., Au_I_ and/or M@Au_I_). The least sensitive locations were those furthest from the center (i.e., Au_O_ and/or M@Au_O_). For the most sensitive or sensitive locations (C, Au_I_, and/or M@Au_I_), the EMFs for the “antibody–NSE–antibody” dimer were stronger than those for the “antibody–CEA–antibody” dimer.

### 2.3. Comparison between the Theoretical and Experiment Results

Some of the theoretical results were also compared with the experimental results from the nanoparticle-based SERS detection of CEA and NSE. Details of the synthesis of the gold nanoparticles and the M@Au nanoparticles have been described previously [9]. For the immunoassay, the procedures for the conjugation of the nanoparticles with the Raman labels and the antibodies are described in the Supplementary Materials. The simulation results were compared with recent experimental results for some systems. For the detection of CEA and NSE, the NPs were conjugated with two separate Raman labels (see Figure S3 in the Supplementary Materials), MBA (4-mercaptobenzoic acid) and DTNB (5,5’-Dithiobis(2-nitrobenzoic acid)), CEA- and NSE-specific detection and capture antibodies, respectively. One example involved the use of the bio-conjugate Au and M@Au NPs in the SERS detection of CEA and NSE (see Supplementary Materials). The experimental SERS intensities were used for the comparison with the calculated EMFs.

**Figure 8:**
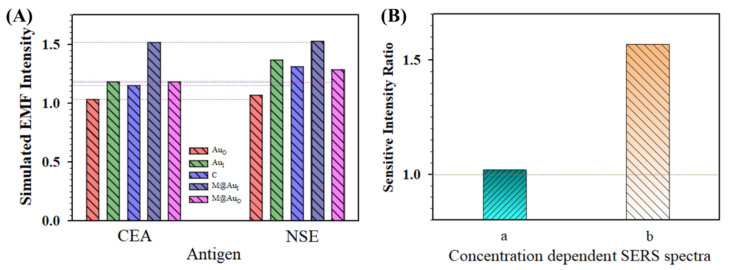
(**A**) Comparison of the simulated EMF intensity for the bioconjugates in detection of CEA and NSE for the dimer of “Ab1/Au_60 nm_–CEA or NSE–Ab2/M_6 nm_@Au_10 nm_ NP”. (**B**) Comparison of the relative sensitivities in terms of intensity ratios of NSE/CEA obtained experimentally from concentration-dependent SERS spectra. (a) Based on the plot of concentration *C* (g/mL) vs. SERS intensity of NSE (Raman label of DTNB, SERS intensity at 1564 cm^−1^) and CEA (Raman label of MBA, SERS intensity at 1595 cm^−1^), see also Figure S3 in the Supplementary Materials; (b) based on the plot of Log*C* (g/mL) vs. SERS intensity of NSE (Raman label of DTNB, SERS intensity at 1331 cm^−1^) and CEA (Raman label of MBA, SERS intensity at 1589 cm^−1^) (see text for details).

Figure 8A summarizes the simulated EMFs for the main locations (Au_O_, Au_I_, C, M@Au_I_, and M@Au_O_) in the dimer of “Ab1/NP_60 nm_–(CEA or NSE)–Ab2/M_6 nm_@Au_10 nm_ NP”. Overall, the EMF intensities for all five locations for the NSE case were greater than those in the CEA case. In Figure 8B(a), the relative sensitivity in terms of the intensity ratio of NSE/CEA obtained from concentration-dependent SERS spectra is clearly greater than 1.0, showing a clear agreement between the EMFs and the experiment data of the bioconjugates in detection of CEA and NSE (see related experimental details in the Supplementary Materials). In the presence of CEA or NSE, the SERS spectra were obtained for the bio-conjugates of MNP@Au NP and Au NPs that were conjugated with the capture/detection antibodies.

Figure 8B also compares the data with the intensity ratio from the experimental result obtained using a similar strategy but a very different nanoparticle conjugation approach (b) [22]. Flower-like gold nanoparticles (~748 nm) were conjugated with anti-CEA antibody and anti-NSE antibody separately, and gold-functionalized magnetic Fe_3_O_4_ nanoparticles (~160 nm) were conjugated with both anti-CEA and anti-NSE antibodies. In other words, while the gold nanoflowers were labeled with different Raman molecules (MBA and DTNB) and antigen-specific antibodies, the SERS-active magnetic nanoparticles were labeled with mixed antibodies. A specific SERS-based immunoassay was used for the simultaneous detection of CEA and NSE. In this case, the result extracted from the NSE/CEA intensity ratio was much greater than 1 (Figure 8B(b)). This result also shows a good agreement between the EMFs and the experiment data of the bioconjugates in the detection of CEA and NSE. The EMF for NSE case was greater than that for the case of CEA. Note that the difference observed in Figure 8B was largely due to the particle size and concentration as well as the subtle difference in antigen–antibody conjugation between our experiment [9] and the report [22]. The density of the particles for flower-like gold nanoparticles (Au NFs, 748 ± 60 nm) prepared for SERS immunoassays detection of CEA and NSE [22] was estimated at ~1.78 × 10^9^ NPs/cm^2^. For the combination of the flower-like gold nanoparticles and gold-coated magnetic nanoparticles (GMNPs, ~160 nm), the particle density was estimated to be ~1.07 × 10^10^ NPs/cm^2^. In comparison, the total particle density for our system with NiFe@Au NP and Au NP was approximately 2.1 × 10^9^ NPs/cm^2^. Apparently, the particle density reported for “(Ab1/Ab2)/Au NF–CEA or NSE–(Ab1/Ab2)/GMNP” [22] was ~5 times higher than our “Ab1/Au NP_60 nm_–CEA or NSE–Ab2/M_6 nm_@Au_10 nm_ NP” in the SERS detection, which explains the difference shown in Figure 8B.

## 3. Conclusions

In summary, a series of dimeric nanoprobes were examined in terms of nanoparticle sizes, core–shell sizes, and interparticle spacing. The results revealed that plasmonic field enhancement not only depends on the particle sizes but also strongly on the relative magnetic core size, Au shell thickness, and the interparticle spacing. The most interparticle distance-sensitive location was the particle edge-to-edge center (C), which was followed by those locations closest to the center. For the most sensitive or sensitive locations (C, Au_I_, and/or M@Au_I_), the EMFs for the “antibody–NSE–antibody” dimer were stronger than those for the “antibody–CEA–antibody” dimer, which was confirmed by experimental data from SERS detection of the cancer biomarkers CEA and NSE. The results have implications for the design of functional nanoprobes for built-in nanogap-based multiplex detection [1,30] and optimization of SERS-based biosensors for detection of cancer biomarkers, which is part of our ongoing work. In an earlier study of nanogap-based multiplex detection [30], self-assembled arrays of porous AuAg nanoparticles were prepared as built-in nanogaps for highly sensitive SERS detection of organic dyes (e.g., Rhodamine 6 G). The multiple nanogaps between the nano-granules presented porosities and high surface-to-volume ratios that were exploited for the enhancement of an electromagnetic field at the dense built-in nanogaps, presenting a potential pathway towards creation of SERS hotspots.

## Data Availability

Not applicable.

## Supplementary Materials

The following are available online at https://www.mdpi.com/article/10.3390/s21248345/s1, Related Experimental details and simulation details, Figure S1: Plots of average EMFs near y = 0 (±5 nm) vs. interparticle distance for the dimer of “Ab1/Au_60 nm_–antigen–Ab2/M–core_6 nm_@Au_5 nm_ NP” at the different locations of Au_O_, Au_I_, C, M@Au_I_, and M@Au_O_; Figure S2: Plots of EMFs at y = 0 or near y = 0 (±5 nm) vs. interparticle distance for the sandwich complex of “Ab1 conjugated Au NP_60 nm_–antigen–Ab2 conjugated Au NP_16 nm_ or Au NP_60 nm_” binding at the different locations of Au_O_, Au_I_, C, control-Au_I_, and control-Au_O_; Figure S3: SERS spectra of sandwich bio-conjugates of NiFe@Au and Au NPs conjugated with capture and detection antibodies in response to the addition of CEA (Raman label of MBA) or NSE (Raman label of DTNB) lung cancer biomarkers [31,32,33].
