# Supplementary material for: Assessing Plasmonic Nanoprobes in Electromagnetic Field Enhancement for SERS Detection of Biomarkers
Source: Sensors (Basel). 2021 Dec 14;21(24):8345. doi: 10.3390/s21248345 (PMC8706705; doi:10.3390/s21248345)
Supplement: Supplementary file 1 [file sensors-21-08345-s001.zip › sensors-1427537-supplementary.pdf]

Supplementary Materials

Assessing Plasmonic Nanoprobes in Electromagnetic Field Enhancement for SERS Detection of Biomarkers

Han-Wen Cheng [1,2,*], Shuyan Xue [1], Jing Li [2], Justine S. Gordon [2], Shan Wang [2], Nina R. Filippone [2], Quang Minh Ngo [3,4], and Chuan-Jian Zhong [2,*]

[1] Laboratory of Advanced Materials, Department of Materials Science, Fudan University, Shanghai 200438, P. R. China
[2] Department of Chemistry, State University of New York at Binghamton, Binghamton, NY13902, USA
[3] University of Science and Technology of Hanoi, Vietnam Academy of Science and Technology, 18 Hoang Quoc Viet, Cau Giay, Hanoi, Vietnam
[4] Institute of Materials Science, Vietnam Academy of Science and Technology, 18 Hoang Quoc Viet, Cau Giay, Hanoi, Vietnam
* Correspondence: To whom correspondence should be addressed. E-mail: cjzhong@binghamton.edu (C.J.Z.); hwcheng@binghamton.edu (H.W.C)

Related Experimental and Simulation details

Gold nanoparticles and magnetic core-gold shell nanoparticles. The chemicals used for the synthesis included tetrachlorohydrogenaurate trihydrate ($HAuCl_4 \cdot 3H_2O$, 99%), sodium citrate (Cit, 99%), hydrazine hydrate ($H_2NNH_2 \cdot H_2O$), Nickel(II) chloride ($NiCl_2$), and ferrous (II) sulfate ($FeSO_4 \cdot 7H_2O$, 99.5%). Other chemicals included glycol ethylene ($HOCH_2CH_2OH$), bovine serum albumin (BSA), 4-mercaptobenzoic acid (MBA), and 5,5′-Dithiobis(2-nitrobenzoic acid) (DTNB, 99%). These were purchased from Sigma-Aldrich. Human carcinoembryonic antigen (CEA), antihuman CEA monoclonal antibodies from mouse (capture antibody (Ab1), clone # M111147, and detection antibody (Ab2), clone # M111146), Human neuron-specific enolase (NSE), antihuman NSE monoclonal antibodies (NSE capture antibody), antihuman NSE monoclonal antibodies (NSE detection antibody, clone # M32234, Fitzgerald Industries International, Inc) were purchased from Fitzgerald Industries International, Inc. Other chemicals including sodium hydroxide, sodium chloride, potassium chloride, borate buffer, and phosphate buffer were purchased from Fisher Scientific or Alfa Aesar. All chemicals were used as received. Deionized water (DI water) was purified with a Millipore Milli-Q water system (18.2 M) and sterilized at 120 ^{o}C for 30 mins for biomarker detection experiments. The synthesis of NiFe core-Au shell nanoparticles (NiFe@Au) involved an initial synthesis of NiFe seed nanoparticles and successive reduction of $HAuCl_4$ by seeded growth method.[S1, S2] NiFe seed nanoparticles were synthesized by hydrothermal methods.[S1] For example, typically, 0.783 mmol of $FeSO_4 \cdot 7H_2O$ and 0.391 mmol of $NiCl_2$ were firstly dissolved in 100 mL milliQ water followed by adding 0.291 mmol sodium citrate as the capping agents. Then the pH in the mixture solution was adjusted to 6.4 by 1.00 M NaOH before adding 4 mL hydrazine hydrate and 1.8 mL ethylene glycol. The final

mixture solution was stirred mechanically under N_2 gas for 1 hr before transferring to autoclave at 135 ^{o}C for 19 hrs. The final NiFe seed products were then collected and cleaned by magnetic bar four times achieving pH = 7. The resultant NiFe magnetic nanoparticles were redispersed in milliQ water and stored at room temperature for further use. The average size for the as-synthesized NiFe seeds was about 6 nm. NiFe@Au nanoparticles were prepared by a modified seeded growth method reported previously.[S2] Briefly, 2 mL NiFe seeds were added to $HAuCl_4$ solution with controlled concentration before adjusting pH to 7.2 by 0.10 M NaOH solution. Sodium acrylate was then added achieving a pH of around 7.8. The resultant mixture solution was sealed, kept stirring under dark atmosphere for three days. In the end, the final products in red color were cleaned by magnetic bar and redispersed in miliQ water three times before further use, which showed an average size of 27 nm with uniform size distribution. Gold nanoparticles (60-nm) were synthesized following a seeded growth protocol, reported previously.[S2,S3] Briefly, Au seeds were synthesized refluxing a mixture solution of HAuCl4 (0.5 mM) with acrylate (1 mM) for 30 mins. The seeds then underwent a seeded growth reaction in the presence of HAuCl4 under controlled concentrations of the reducing and capping agents to form 60 nm Au NPs. Details of the synthesis were described in our previous report.[S4]

Conjugation with Raman labels and antibodies. The preparation of bio-conjugates of M@Au nanoparticles followed the protocol reported previously.[S5] Briefly, the M@Au nanoparticles were collected by centrifugation, followed by dispersing in borate buffer. CEA or NSE capture antibody were mixed with M@Au nanoparticles and incubated under ambient atmosphere for 2 hrs. After centrifugation and resuspension in borate buffer, BSA (5%) was added to block active sites between antibodies. The resulting bio-conjugates were cleaned by centrifugation and stored at 4 ^{o}C for further use. Two types of SERS tags were prepared for simultaneous detection of CEA and NSE. 5.0 μL of 1.0 mM MBA or DTNB Raman labels were added to Au nanoparticles respectively, followed by overnight shaking. The MBA or DTNB modified Au nanoparticles were purified at 10000 rpm for 5 mins for 3 times. After purification, the precipitates were dispersed in 10 mM PBS buffer (pH = 7.4). 5 μL of 25 mM EDC and 5 μL of 25 mM NHS were added to activate the − COOH groups on the surface of gold nanoparticles. Excess EDC and NHS were removed by centrifugation (10000 rpm, 5 mins). Then anti-CEA or anti-NSE antibodies were added to react with the activated carboxyl groups on Au nanoparticles at 25 °C for 2 h. The mixtures were centrifuged 3 times to remove excess antibodies (10000 rpm, 5 mins). Purified SERS tags were resuspended in 10 mM PBS buffer solution (pH 7.4) and stored at 4 ^{o}C for further use. Details of the bioconjugation were described in our previous report.[S4]

SERS detection. SERS spectra were recorded on a Thermo Scientific DXR™ Raman microscope equipped with 780 nm laser source. The laser power is 8 mW with laser aperture of 50 μm slit and the focal spot for laser excitation is estimated to be 3.1 μm. All spectra were collected in the range of 350-3389 cm^{-1}, with exposure number of 10 and exposure time of 15 sec.

Simulation. Simulations of the localized surface plasmon resonance and electrical intensity were computed using MNPBEM toolbox,[S6] which incorporates the dielectric constants and refractive indices of Au and Fe_3O_4 for NP boundaries in aqueous environment at a laser wavelength of 780 nm.[S2,S7,S8] The simulation involved setting up the nanoparticles' boundary conditions and the dielectric environment to calculate the optical absorption and the electrical field around the nanoparticles.

Supplementary Figures:

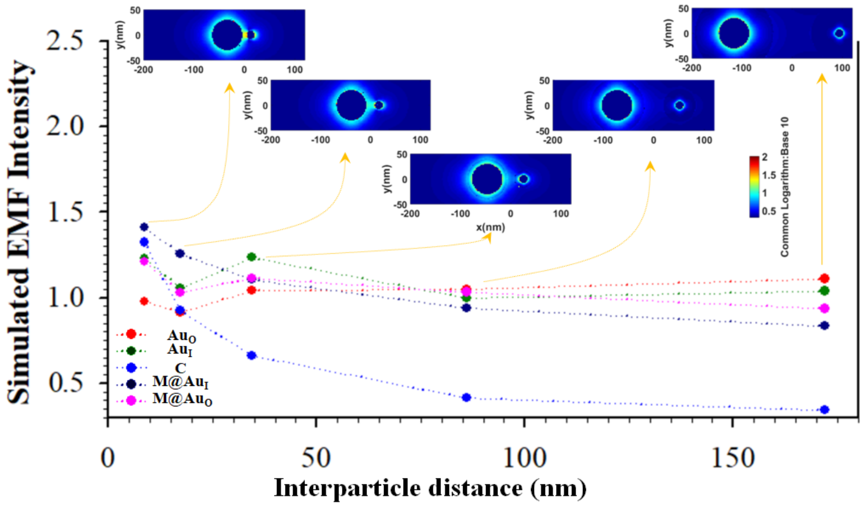

Figure S1. Plots of average EMFs near y=0 (±5 nm) *vs*. interparticle distance for the dimer of "Ab1/$Au_{60\,nm}$ -antigen-Ab2/M-$core_{6\,nm}$@$Au_{5\,nm}$ NP" at the different locations of Au_O, Au_I, C, M@Au_I and M@Au_O. Inset: plasmonic field simulation results in terms of 2D plotting.

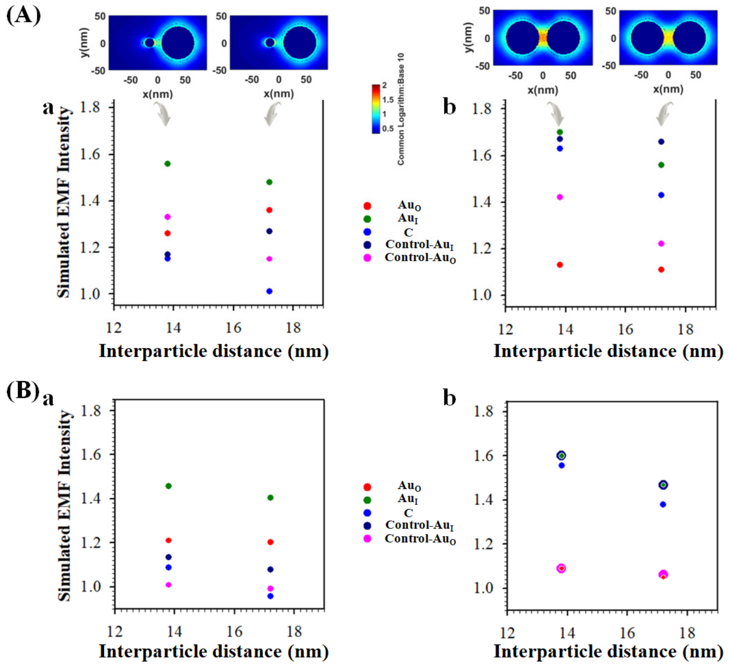

Figure S2. Plots of EMFs at y=0 (A) or near y=0 (±5 nm) (B) *vs*. interparticle distance for the sandwich complex of "Ab1 conjugated Au $NP_{60\,nm}$ – antigen –Ab2 conjugated Au $NP_{16\,nm}$ (a) or Au $NP_{60\,nm}$ (b)" binding at the different locations of Au_O, Au_I, C, control-Au_I and control-Au_O. Inset: plasmonic field simulation results in terms of 2D plotting.

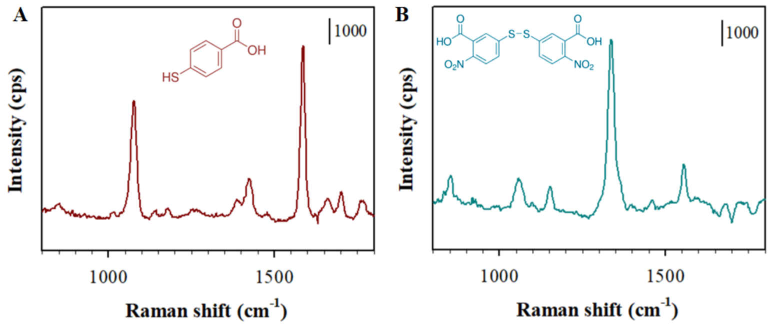

Figure S3. SERS spectra of sandwich bio-conjugates of NiFe@Au and Au NPs conjugated with capture and detection antibodies, in response to the addition of CEA (A, Raman label of MBA) or NSE (B, Raman label of DTNB) lung cancer biomarkers.

References

1 Liu, Y.; Chi, Y. X.; Shan, S. Y.; Yin, J.; Luo, J.; Zhong, C. J. Characterization of Magnetic NiFe Nanoparticles with Different Bimetallic Composition. *J. Alloy Compd.* **2014**, *587*, 260-266.

2 Njoki, P. N.; Lim, I. I. S.; Mott, D.; Park, H. Y.; Khan, B.; Mishra, S.; Sujakumar, R.; Luo, J.; Zhong, C. J. Size Correlation of Optical and Spectroscopic Properties for Gold Nanoparticles. *J. Phys. Chem. C* **2007**, *111*, 14664-14669.

3 Crew, E.; Yan, H.; Lin, L.; Yin, J.; Skeete, Z.; Kotlyar, T.; Tchah, N.; Lee, J.; Bellavia, M.; Goodshaw, I.; Joseph, P.; Luo, J.; Gal, S.; Zhong, C. J. DNA Assembly and Enzymatic Cutting in Solutions: A Gold Nanoparticle Based SERS Detection Strategy. *Analyst* **2013**, *138*, 4941-4949.

4 Li, J.; Skeete, Z.; Shan, S.; Yan, S.; Kurzatkowska, K.; Zhao, W.; Ngo, Q. M.; Holubovska, P.; Luo, J.; Hepel, M.; Zhong, C. J. Surface Enhanced Raman Scattering Detection of Cancer Biomarkers with Bifunctional Nanocomposite Probes. *Anal Chem*. **2015**, *87*, 10698-10702

5 Chen, S. A.; Yuan, Y. X.; Yao, J. L.; Han, S. Y.; Gu, R. A. Magnetic separation and immunoassay of multi-antigen based on surface enhanced Raman spectroscopy. *Chem Commun* **2011**, *47*, 4225-4227.

6 Hohenester, U.; Trügler, A. MNPBEM - A Matlab Toolbox for the Simulation of Plasmonic Nanoparticles. *Comput. Phys. Commun.* **2012**, *183*, 370–381.

7 Skeete, Z.; Cheng, H.-W.; Ngo, Q. M.; Salazar, C.; Sun, W.; Luo, J.; Zhong, C. J. "Squeezed" Interparticle Properties for Plasmonic Coupling and SERS Characteristics of Duplex DNA Conjugated/Linked Gold Nanoparticles of Homo/Hetero-Sizes. *Nanotechnology* **2016**, *27*, 325706.

8 Skeete, Z.; Cheng, H. W.; Li, J.; Salazar, C.; Sun, W.; Ngo, Q. M.; Lin, L.; Luo, J.; Zhong, C. J. Assessing Interparticle Spatial Characteristics of DNA-Linked Core−Shell Nanoparticles with or without Magnetic Cores in Surface Enhanced Raman Scattering. *J. Phys. Chem. C* **2017**, *121*, 15767−15776.
